# Gastrointestinal Carriage Is a Major Reservoir of *Klebsiella pneumoniae* Infection in Intensive Care Patients

**DOI:** 10.1093/cid/cix270

**Published:** 2017-03-24

**Authors:** Claire L. Gorrie, Mirjana Mirčeta, Ryan R. Wick, David J. Edwards, Nicholas R. Thomson, Richard A. Strugnell, Nigel F. Pratt, Jill S. Garlick, Kerri M. Watson, David V. Pilcher, Steve A. McGloughlin, Denis W. Spelman, Adam W. J. Jenney, Kathryn E. Holt

**Affiliations:** 1 Department of Biochemistry and Molecular Biology, Bio21 Molecular Science and Biotechnology Institute and; 2 Department of Microbiology and Immunology at the Peter Doherty Institute for Infection and Immunity, The University of Melbourne, and; 3 Microbiology Unit, Alfred Health, Melbourne, Victoria, Australia;; 4 Wellcome Trust Sanger Institute, Hinxton, Cambridgeshire, United Kingdom;; 5 Infectious Diseases Clinical Research Unit and; 6 Intensive Care Unit, The Alfred Hospital,; 7 Australian and New Zealand Intensive Care – Research Centre, School of Public Health and Preventive Medicine, Monash University, and; 8 Microbiology Unit & Department of Infectious Diseases, The Alfred Hospital, Melbourne, Victoria, Australia

**Keywords:** *Klebsiella pneumoniae*, gastrointestinal colonization, genomic epidemiology, intensive care, hospital acquired infection.

## Abstract

**Background.:**

*Klebsiella pneumoniae* is an opportunistic pathogen and leading cause of hospital-associated infections. Intensive care unit (ICU) patients are particularly at risk. *Klebsiella pneumoniae* is part of the healthy human microbiome, providing a potential reservoir for infection. However, the frequency of gut colonization and its contribution to infections are not well characterized.

**Methods.:**

We conducted a 1-year prospective cohort study in which 498 ICU patients were screened for rectal and throat carriage of *K. pneumoniae* shortly after admission. *Klebsiella pneumoniae* isolated from screening swabs and clinical diagnostic samples were characterized using whole genome sequencing and combined with epidemiological data to identify likely transmission events.

**Results.:**

*Klebsiella pneumoniae* carriage frequencies were estimated at 6% (95% confidence interval [CI], 3%–8%) among ICU patients admitted direct from the community, and 19% (95% CI, 14%–51%) among those with recent healthcare contact. Gut colonization on admission was significantly associated with subsequent infection (infection risk 16% vs 3%, odds ratio [OR] = 6.9, *P* < .001), and genome data indicated matching carriage and infection isolates in 80% of isolate pairs. Five likely transmission chains were identified, responsible for 12% of *K. pneumoniae* infections in ICU. In sum, 49% of *K. pneumoniae* infections were caused by the patients’ own unique strain, and 48% of screened patients with infections were positive for prior colonization.

**Conclusions.:**

These data confirm *K. pneumoniae* colonization is a significant risk factor for infection in ICU, and indicate ~50% of *K. pneumoniae* infections result from patients’ own microbiota. Screening for colonization on admission could limit risk of infection in the colonized patient and others.

## Summary

*Klebsiella pneumoniae* colonization is a significant risk factor for infection in ICU, with approximately half of *K. pneumoniae* infections resulting from patients’ own microbiota. Screening for colonization on admission could limit risk of infection in the colonized patient and others.

The emergence of multidrug-resistant (MDR) *Klebsiella pneumoniae* has resulted in a dramatic increase in research into reservoirs and risk factors for healthcare-associated (HA) *K. pneumoniae* infections, largely focused on extended spectrum beta-lactamase (ESBL) or carbapenemase-producing (CP) bacteria isolated from infections and intrahospital outbreaks [1–3]. These studies have demonstrated transmission of ESBL or CP *K. pneumoniae* between patients and show that gastrointestinal (GI) tract colonization with ESBL or CP *K. pneumoniae* can be a risk factor for infection [3, 4]. Yet although the majority of *K. pneumoniae* HA infections are not ESBL or CP [5, 6], there are little data on the frequency and clinical relevance of colonization with *K. pneumoniae* more generally.

*Klebsiella pneumoniae* is known to asymptomatically colonize the skin, mouth, respiratory and GI tracts, although few studies address this specifically. Using culture-free methods, *K. pneumoniae* was detected in approximately 10% of Human Microbiome Project samples from the mouth, nares, and skin, and 3.8% of stool samples [7]. Using bacteriological culture, a 2010 study detected nasopharyngeal carriage in 15% of Indonesian adults and 7% of children [8], whereas a 2014 study detected nasopharyngeal carriage in 2.7% of Vietnamese adults and throat carriage in 14% [9].

Patients suffering from CP *K. pneumoniae* infections (typically ST258), or from pyogenic liver abscess caused by hypervirulent *K. pneumoniae* (ST23), have been shown to carry their infecting strain in their GI tract for between 30 days (≤74%) and 6 months (<30%) following discharge from hospital [10]. However, the question of whether *K. pneumoniae* colonization on admission to hospital poses a risk for subsequent infection is much less clear. A 1971 study found 18.5% of patients admitted to various wards in the Denver Veterans Administration Hospital were culture-positive for rectal carriage of *K. pneumoniae*, and carriage was significantly associated with risk of subsequent HA infection (45% vs 11%) [11]. A 2016 study at the University of Michigan Health System tertiary care hospital reported similar colonization rates (23%) and increased risk of infection following colonization (5.2% in colonized vs 1.3% in noncolonized) [12].

Here, we assessed the prevalence of *K. pneumoniae* colonization in an at-risk cohort in an intensive care unit (ICU) within a modern, well-equipped, and well-managed tertiary teaching hospital in Australia. Additionally, we investigated whether colonization on admission enhances risk of subsequent *K. pneumoniae* infection among ICU patients and the relative contribution of patients’ own gut microbiota and intra-hospital transmission to the burden of *K. pneumoniae* carriage and infection in the ICU.

## METHODS

### Ethics

Ethical approvals for these studies were granted by the Alfred Hospital Ethics Committee (project numbers 550/12 and 526/13).

### Recruitment and Specimen and Data Collection

The *Klebsiella* Acquisition Surveillance Project at Alfred Health (KASPAH) was conducted from April 1, 2013, to March 31, 2014. Eligible patients (adults aged ≥18 years and expected to spend ≥3 days in ICU) were recruited as soon as possible after admission, and baseline rectal and throat screening swabs were collected. For the first 9 months verbal consent was required to participate. For the last 3 months a universal surveillance study for multidrug resistant organisms was conducted for which consent was waived; samples and data from this period were also included in KASPAH (see Supplementary Methods and Supplementary Figure 1). Follow-up swabs were repeated each 5–7 days after baseline for the duration of ICU stay and up to 4 days following transfer to another ward. Information on age, sex, dates of hospital and ICU admission/s, surgery in the last 30 days, and antibiotic treatment in the last 7 days were extracted from hospital records at the time each swab was taken. Dates of discharge and/or death were extracted from hospital records at the conclusion of the study. All clinical isolates recovered from ICU patients and identified as *K. pneumoniae* infections by the hospital diagnostic laboratory as part of routine care were included in the study. Full details are given in Supplementary Methods.

### Community Associated vs Healthcare Associated Carriage

Individuals in the community-associated (CA) screening group include patients who were both (i) admitted to the Alfred Hospital ICU either directly (day 0) or via another ward on day 0, 1, or 2 of the original hospital admission; and (ii) first swabbed on day 0, 1, or 2 of that admission. Patients first swabbed on day 3 or later of their hospital admission are included in the HA/Day 3+ screening group. Individuals referred to the Alfred Hospital ICU by the trauma ward of another hospital were assumed to be emergency admissions from the community and were assigned to the CA/Day 0–2 or HA/Day 3+ screening groups according to the day of first swab relative to their Alfred Hospital admission. All other patients transferred from another hospital were included in the HA/D3+ screening group. As such, the CA/Day 0–2 screening groups represent individuals admitted directly to the hospital from the community, whereas the HA/D3+ group includes individuals with recent hospital exposure.

### DNA Extraction and Sequencing

DNA was extracted from overnight cultures using a phenol:chloroform protocol and phase lock gel tubes and sequenced via Illumina HiSeq to generate 125 bp paired-end reads (see Supplementary Methods). Following quality control checks of the sequence data, 148 isolates from 106 patients were subjected to comparative genomic analysis (see Supplementary Table 1 for genome sequence accessions). A maximum likelihood phylogenetic tree was inferred from an alignment of all single-nucleotide polymorphisms (SNPs) identified within core *K. pneumoniae* genes using FastTree v2.1.8 [13, 14]. Lineages were defined based on this tree using RAMI [15] and multilocus sequence types were assigned using SRST2 [16]. Isolates falling within the same lineage were further investigated to identify pairwise SNPs via assembly and read mapping; full details of genomic analyses are given in Supplementary Methods.

### Statistical Analysis

All statistical analyses were conducted using R (v3.3.1) (details in Supplementary Methods).

## RESULTS

### **Klebsiella pneumoniae** Carriage

A total of 498 patients expected to spend ≥3 days in ICU were recruited and screened for *K. pneumoniae* carriage. This represents 33% of eligible patients during the consent-based recruitment period and 75% in the universal surveillance period (18% of all ICU admissions in the study period). Fifty-four patients (10.8%) tested positive at baseline screening (50 GI carriage only, 2 throat carriage only, 2 both). Carriage was detected at the same frequency in males and females (11.0% vs 10.4%; *P* = .9), and the median age of carriers was moderately higher than that of carriage-negative participants (67 vs 58 years, Supplementary Figure 2; *P* = .06, Wilcoxon rank-sum test).

We estimated the rate of CA *K. pneumoniae* GI carriage, among patients recruited and swabbed in the ICU within 2 days of their first recorded admission to the Hospital (CA/D0-2 group), to be 5.9% (95% confidence interval [CI], 3% – 8%, Table 1, Supplementary Table 2). The HA GI carriage rate, among patients who were first swabbed in the ICU on or after the third day of admission to the Alfred hospital or following referral from another hospital (HA/D3+ group, Supplementary Figure 3), was significantly higher at 19% (95% CI, 13.6% – 25.7%, odds ratio [OR] = 3.75, *P* = .00001).

**Table 1.** *Klebsiella pneumoniae* GI Carriage Detected at Baseline and Followup Screening of Alfred Hospital Patients

Baseline Carriage Status	Number of Patients (%)	MDR Isolates (%)	Follow-up +ve (%)	Follow-up MDR (%)
CA/Day 0–2	324			
Baseline *Kp* +ve	19 (5.9%)	0 (0%)	2/4 (50%)	0 (0%)
Baseline *Kp* −ve	305 (94.1%)	…	14*/96 (14.6%)	0 (0%)
HA/Day 3+	174			
Baseline *Kp* +ve	33 (19%)	6 (17.6%)	5/13* (38.5%)	2 (40%)
Baseline *Kp* −ve	141 (81%)	…	5/57 (8.8%)	2 (40%)
Total baseline *Kp* +ve	52 (10.4%)	6 (11.3%)	7/17 (41.2%)	2 (28.6%)
Total baseline *Kp* −ve	446 (89.6%)	…	19/153 (12.4%)	2 (10.5%)
Total	498	6 (1.2%)	26/170 (15.3%)	4 (15.4%)

Patient groups: ICU CA/Day 0–2, rectal screening swab obtained on day 0, 1, or 2 of admission to Alfred Hospital and not referred from another hospital (except from trauma unit); ICU HA/Day 3+, rectal screening swab obtained on day 3 or later of admission to Alfred Hospital or referred from another hospital. *These indicate 2 patients from whom swabs yielded isolates that were identified in the hospital laboratory as *K. pneumoniae*, but sequencing of subcultures identified substantial *E. coli*, indicating likely presence of both species.

Abbreviations: GI, gastrointestinal; ICU, intensive care unit; MDR, multidrug-resistant.

One third of the participants in the ICU screening study (n = 170) contributed 1 or more follow-up screening swabs (Table 1). The overall GI carriage rate at follow-up was 15.3% (n = 26/170), similar to the HA GI carriage rate of 19% (OR = 1.3; 95% CI, 0.71–2.38). Participants testing positive on follow-up rectal swabs included 19 who tested negative for *K. pneumoniae* on their baseline rectal swab, yielding a conversion rate of 12%.

None of the 19 CA baseline carriage isolates were MDR (Table 1). GI carriage of MDR strains was detected at similar rates among HA baseline isolates (18%, including 4 ESBL and 2 CP isolates, all in patients who had received antibiotics in the last 7 days) and follow-up screening isolates (16% of patients, including 4 with ESBL and 1 with CP isolates). A total of seven patients contributed both baseline and follow-up GI carriage isolates (Table 1). For 5/7 patients the resistance profiles for the follow-up isolate remained the same as the baseline isolate, and for 2/7 patients the follow-up isolate was more resistant (see Supplementary Tables 1 and 3).

### GI Colonization Is a Source of *Klebsiella* Infection

A total of 49 patients (1.8% of all adult ICU admissions) who spent time in the ICU during their hospital stay were identified as having *K. pneumoniae* infections (11 ESBL, of which 3 were also CP). Most (n = 38) were in the ICU when the *K. pneumoniae*–positive diagnostic specimen was taken, and 10 were in another ward shortly after transfer from ICU. Pneumonia was the most frequent form of *K. pneumoniae* infection in ICU patients (60%), followed by wound infections (15%), nondisseminated urinary tract infection (UTI) (10%), and bacteremia with sepsis (8%). MDR was most common among wound and blood isolates (≥50%) (Table 2).

**Table 2.** Patients With Infection(s) and Time in the ICU

	Pneumonia	UTI (Non-invasive)	Wound	Other	Bacteremia With Sepsis	Total
Recipient in transmission chain	2	1	2	0	1	6
Donor in transmission chain	3	0	1	0	0	4
Prior GI colonization	6	1	1	0	0	8
Prior throat colonization	1	0	0	0	0	1
Unknown source (unique lineage)	16 (12)	3 (3)	4 (1)	3 (2)	3 (1)	29
**Total**	**28** **(5 MDR)**	**5**	**8** **(4 MDR)**	**3**	**4** **(2 MDR)**	**48**

Type of infection and source of infection outlined (position/presence in transmission chain, prior colonization, unknown). Note that 3 patients had UTI and bacteremia with sepsis; they are represented here in the bacteremia with sepsis column. “Unknown source” includes those infections for which there is no genetic or epidemiological evidence to indicate whether the infection has arisen from a patient’s own carriage strains or through transmission from another source; numbers in brackets indicate the number of such infections associated with a lineage that was unique to that patient. Note that “unknown source” also includes one patient who had a wound infection diagnosed as *K. pneumoniae* but genome sequencing found the subcultured isolate to be dominated by *A. baumannii* DNA; this is consistent with mixed infection or contamination, and prevents reliable comparative analysis with other *K. pneumoniae* strains.

Abbreviations: GI, gastrointestinal; ICU, intensive care unit; MDR, multidrug-resistant; UTI, urinary tract infection.

**Figure 1.**
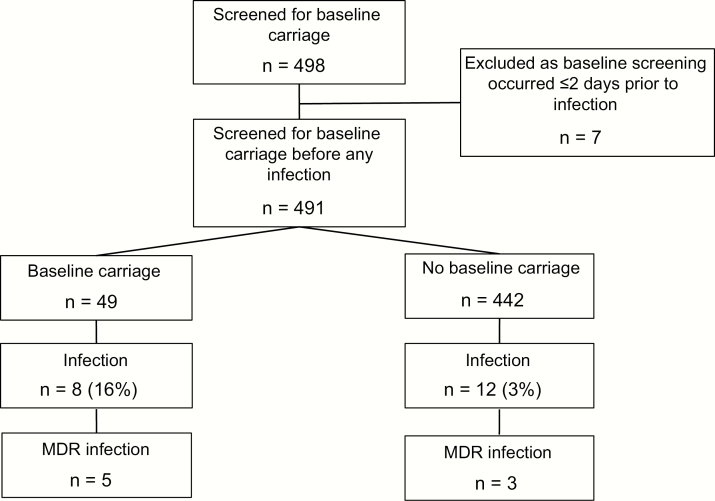
Flowchart outlining number of patients included in each part of carriage rates analyses.

To assess whether *K. pneumoniae* GI carriage on admission to ICU was a risk factor for subsequent *K. pneumoniae* infection during hospital stay, we examined the subset of 491 individuals whose baseline screening swab was obtained at least 2 days prior to collection of any clinical specimen from which *K. pneumoniae* was isolated (Figure 1). The rate of *K. pneumoniae* infection was significantly higher among patients who were culture-positive for GI carriage at baseline compared to those who were culture-negative (16% vs 3%; OR = 6.9, 95% CI, 2.3 – 19.7, *P* < .001, see Figure 1). Of all ICU patients who developed *K. pneumoniae* infections and contributed baseline screening swabs, 48% (n = 13/27) tested positive for *K. pneumoniae* GI carriage at baseline (including 8 who were screened >2 days prior to developing the infection).

**Figure 2.**
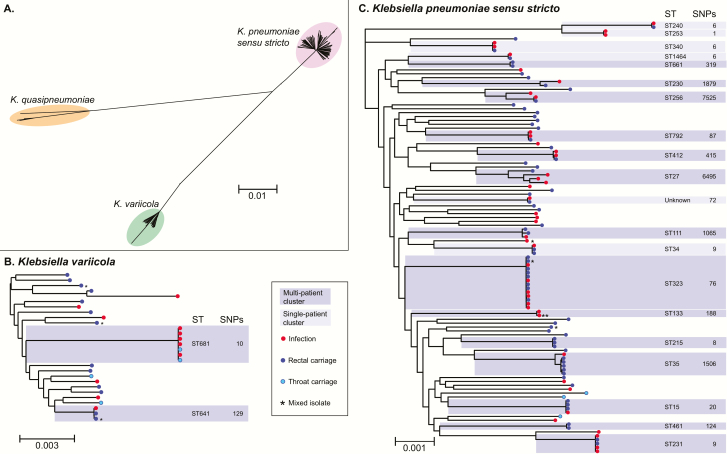
Genome diversity of isolates from ICU patients identified as *Klebsiella pneumoniae*. All trees are maximum likelihood trees inferred from core genome SNP alignments. Scale bars indicate average number of substitutions per site across the genome. Tip colours indicate isolate source as per inset legend. *Possible mixed isolate (0.02–0.1 het/hom SNP ratio, excluded from pairwise SNP analysis in Figure 3). **Clinical isolate from sputum (KC0048), may not represent an infection. Phylogenetic lineages to which more than one ICU isolate belongs are highlighted and labeled with their corresponding multi-locus sequence type (ST) and the total number of SNPs identified between isolates in the lineage; darker shading indicates multiple patients contributed isolates in that cluster, as per inset legend. (*A*), Unrooted tree of all isolates, revealing three distinct species that are typically identified as *K. pneumoniae* in diagnostic laboratories. (*B*), Midpoint rooted species tree for *K. variicola* isolates. (*C*), Midpoint rooted species tree for *K. pneumoniae sensu stricto* isolates. Abbreviations: ICU, intensive care unit; SNP, single-nucleotide polymorphism.

To determine whether infections were caused by patients’ own colonizing bacteria and to identify transmission between ICU patients, we sequenced the genomes of all *K. pneumoniae* isolated from patients who had spent any time in the ICU during their hospital stay(s). A total of 143 high-quality whole genome sequences were obtained from 106 patients, including 56 clinical, 80 GI carriage, and 7 throat carriage isolates. Core genome phylogenetic analysis (Figure 2) revealed the presence of 61 lineages of *K. pneumoniae sensu stricto* (n = 111 isolates) and 24 lineages of 2 closely related species: 20 *K. variicola* lineages (n = 28 isolates) and 4 *K. quasipneumoniae* lineages (n = 4 isolates). The average distance between lineages within each species was 0.5% nucleotide divergence, representing thousands of years of evolutionary separation based on molecular clock estimates for *K. pneumoniae* [17].

Most *Klebsiella* lineages (n = 69/85, 81%) were identified in just 1 patient, and 60% of patients (n = 64) had their own unique lineage not observed in any other patients (Figure 2, Supplementary Table 3). Half the infections (n = 24/48) were caused by a lineage unique to the patient. Fifteen patients had both GI carriage and infection isolates available for genome comparison; 12 of these pairs matched at the lineage level (including 6 patients whose carriage isolate was collected >2 days prior to the infection).

### *Klebsiella* Transmission in the ICU

**Figure 3.**
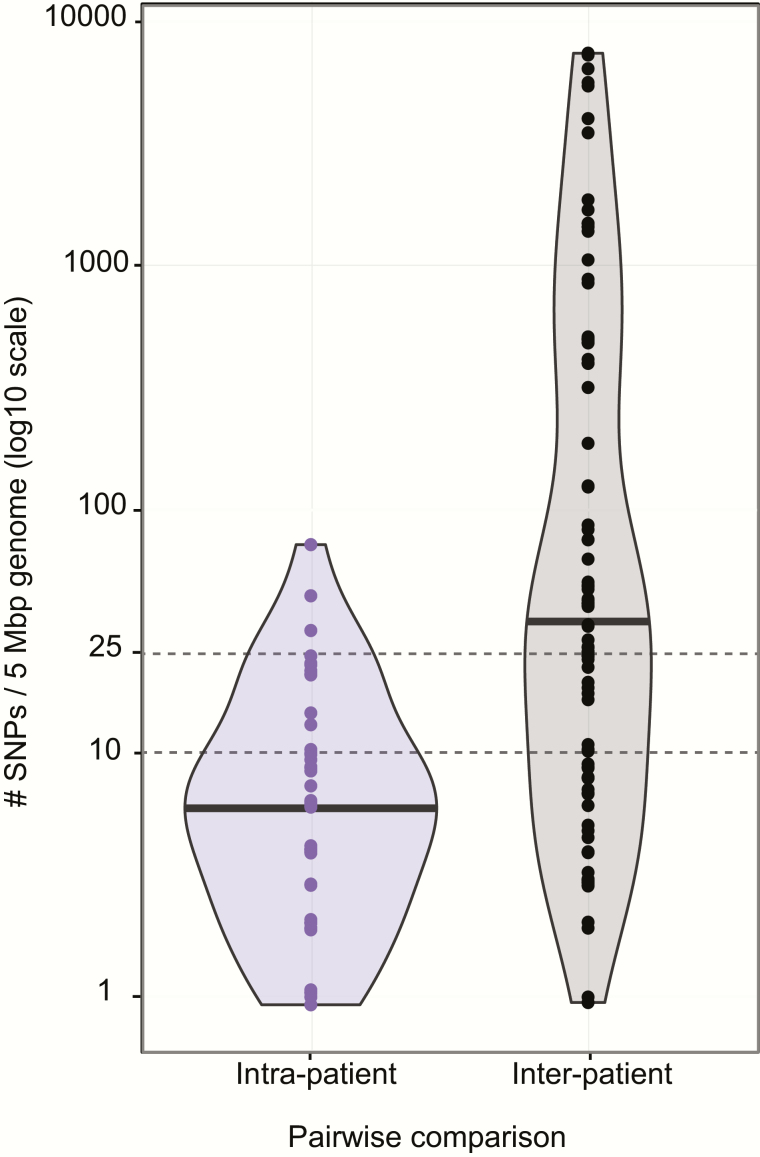
Pairwise genetic distances between isolates belonging to the same lineage, expressed as SNPs per 5 Mbp of genome in order to normalise for differences in shared gene content between strain pairs. Violin plots showing distribution of pairwise genetic distances intra- and inter-patient; black bars indicate the median value. Note the log_10_ scale which excludes display of 1 strain pair that was separated by 0 SNPs. Abbreviation: SNP, single-nucleotide polymorphism.

**Figure 4.**
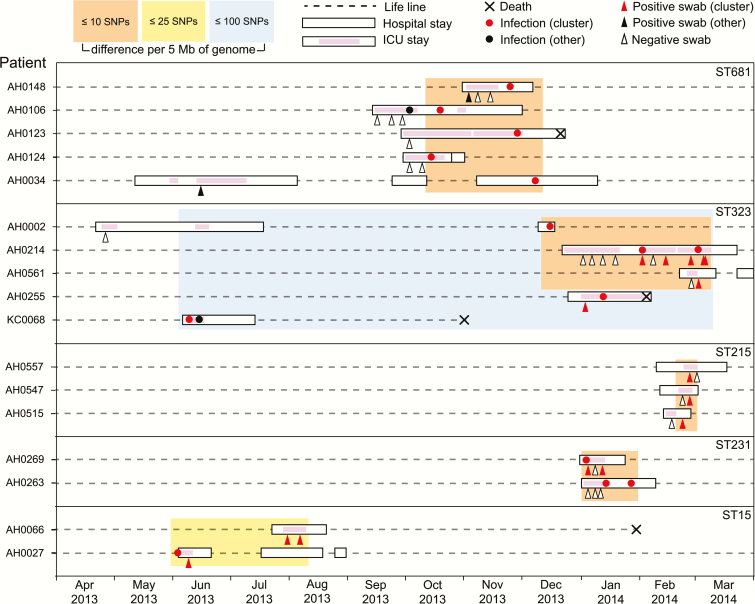
Timelines for all lineages detected in multiple patients that show any inter-patient pairwise genetic distance between isolates of ≤25 SNPs per 5 Mbp. Lineages are boxed and labeled with their multi-locus sequence type (ST). Each horizontal dashed line indicates the time line for a patient, labelled to the left (crosses indicate date of death where applicable). Periods of Alfred Hospital admission are indicated as white boxes, periods in ICU as pink shading. Circles indicate *K. pneumoniae* infection isolates (red, belonging to the lineage; black, other lineage); triangles indicate rectal screening swabs (red, *K. pneumoniae* belonging to the lineage; black, *K. pneumoniae* of another lineage; unfilled, negative for *K. pneumoniae*). Orange boxes indicate groups of isolates for which all patients have at least one pairwise genetic distance of ≤10 SNPs per 5 Mbp with another in the group; similarly for yellow (≤25 SNPs) and blue (≤100 SNPs) boxes. Abbreviations: ICU, intensive care unit; SNP, single-nucleotide polymorphism.

Sixteen *Klebsiella* lineages were detected in more than 1 patient (dark shading, Figure 2). Lineage sharing between patients could result from recent transmission of bacteria within the hospital (strain sharing) or by independent acquisition of a lineage that has been circulating in the community (lineage sharing). To distinguish these possibilities we compared intra-patient and inter-patient pairwise SNP distances (Figure 3). Intra-patient genetic distances were nearly all (97%) less than 25 SNPs per 5 Mbp, and most (82%) were less than 10 SNPs, whereas inter-patient genetic distances ranged from 0 to >5000 SNPs (Figure 3). Using 25 and 10 SNPs per 5 Mbp as cut-offs to indicate likely and very likely strain sharing between patients, we identified 5 groups of ICU patients that likely shared *Klebsiella* strains. Strikingly, each of these groups comprised patients with overlapping admissions, making them epidemiologically plausible intra-hospital transmission chains (Figure 4). For the other 11 lineages that were detected in more than 1 ICU patient, between-patient SNP distances exceeded 80 SNPs per 5 Mbp and ICU admissions were generally nonoverlapping (Supplementary Figure 4). Six infection episodes (n = 6/49, 12%) were attributable to these intra-hospital transmission chains (n = 4 ST681 [*K. variicola*], n = 1 ST323, n = 1 ST231; Figure 4). These included 2 episodes of pneumonia, 2 wound infections, and 2 UTIs, one of which disseminated to cause bacteremia with sepsis (Table 2, Supplementary Table 3). Of the 4 donors in the transmission chains, 3 had pneumonia and 1 had a wound infection. Most of the infections associated with transmission were MDR (3/4 donors and 3/6 recipients), yielding a strong association between MDR infections and transmission in the ICU (OR = 13.6, *P* = .002). In addition, we identified 4 patients whose *K. pneumoniae* carriage was attributable to intra-hospital transmission chains but did not result in any recorded *K. pneumoniae* infection during hospital stay (n = 1 ST323 [MDR], n = 2 ST215 [non-MDR], n = 1 ST15 [MDR]), representing 5% of all carriage-positive patients.

## DISCUSSION

We estimated a 5.9% CA rate for culture-positive GI carriage of *K. pneumoniae*, similar to the 3.9% estimated among healthy individuals in the Human Microbiome Project based on 16S rRNA amplicon sequencing of stool samples [7]. HA carriage among ICU patients was estimated to be much higher at 19%, with 12% of patients converting from culture-negative at baseline to culture-positive on follow-up. This likely reflects acquisition of bacteria in the hospital (transmission of strains was directly observed in 5% of cases) and/or selection for growth of pre-existing *K. pneumoniae* in the GI microbiome during hospitalization (MDR rate was 18% in HA baseline carriage and 15% at follow-up, but MDR was not detected in CA baseline carriage). The HA rate estimated here is similar to the culture-positive GI carriage rates estimated in other hospital studies [11, 12].

Our data demonstrate that *K. pneumoniae* is a fairly common component of the human GI microbiome (5.9%) and of clinical significance in the ICU setting, as: (i) *K. pneumoniae* carriage on admission to ICU was significantly associated with subsequent *K. pneumoniae* infection (OR = 6.9, *P* = .0003), consistent with the results reported from the 1970s Denver study (OR = 4.0, *P* = .0009 [11]) and the 2016 Michigan study (OR = 4.1, *P* = .00002 [12]); and (ii) the WGS data confirmed a direct link between colonizing and infecting strains in 13 patients (80% of those with paired isolates available for testing), also consistent with the Michigan study [12]. We found strong evidence that a large proportion of ICU *K. pneumoniae* infections are attributable to patients’ own GI microbiota: (i) of all 49 *K. pneumoniae* infections diagnosed in ICU patients during the study period, 49% were associated with *K. pneumoniae* lineages unique to the patient; and (ii) of the 27 *K. pneumoniae* infections diagnosed in ICU patients from whom screening swabs were obtained, 48% occurred in patients who tested positive for prior GI colonization with *K. pneumoniae.* In contrast, only 12% of infections showed evidence of resulting from intra-hospital transmission in this setting. This suggests that although measures to reduce cross-contamination between patients are necessary, they are not sufficient to eliminate *K. pneumoniae* infections in hospitalized patients, and measures to minimize the risk of infection with the patients’ own microbiome deserves significant attention [21–23].

Key strengths of this study are the prospective cohort design and the use of WGS to confirm species identification and strain relatedness for all *K. pneumoniae* isolated from ICU patients, regardless of antimicrobial susceptibility. Most previous studies of *K. pneumoniae* colonization in hospitals have focused on ESBL and/or CP isolates only, which do not represent the major burden of *K. pneumoniae* infections. WGS demonstrated that some isolates identified as *K. pneumoniae* by MALDI-TOF actually belonged to closely related groups that have recently been described as separate species [24], *K. quasipneumoniae* and *K. variicola* (Figure 2). Because these species are very closely related to *K. pneumoniae sensu stricto* (~4% nucleotide divergence), are clinically indistinguishable, and are typically identified as *K. pneumoniae* in diagnostic laboratories, comparable studies reporting *K. pneumoniae* carriage encompass the entire *K. pneumoniae* complex. WGS also demonstrated that a small number of isolates identified as *K. pneumoniae* were contaminated to varying degrees with non-*Klebsiella* DNA (n = 6) or multiple *Klebsiella* strains (n = 8) (Supplementary Methods). These may reflect coinfection followed by selection for different subpopulations in the laboratory used for the different tests. These samples were therefore excluded from high-resolution genomic analysis for attribution purposes (Figure 1, Supplementary Table 1) but were included in calculation of overall rates of infection and carriage, which reflect solely the routine laboratory identification and are directly comparable with other reported results.

The main limitations of the study arise from the swabbing procedure to identify *K. pneumoniae* colonization on admission. Efforts were made to collect swabs as soon as possible following admission; however, delays due to obtaining consent and avoiding patient care disruption meant that it was often not possible to obtain screening swabs on the day of admission. Notably, similar culture-positive rates were observed for swabs collected on day 0, 1, or 2 of admission (Supplementary Table 2), so it is unlikely that this had a significant impact on CA colonization results. Although the recruitment rate was low (33%) during the consent-based recruitment period, the demographic features and *K. pneumoniae* colonization rate in this group were not significantly different from those recruited under the universal surveillance protocol (75% recruitment; Supplementary Figure 1); hence, there is no evidence of significant selection bias. We used rectal swab culture to determine *K. pneumoniae* GI colonization status; however, although this approach is standard for pathogen carriage screening [25–27], its sensitivity to detect *K. pneumoniae* is not well characterized and likely depends on the *K. pneumoniae* strain, GI microbiome composition, and recent antimicrobial exposures. There is likely a significant false negative rate. Hence our study probably underestimates the rate of colonization in the community and the contribution of colonisation to subsequent infection. Additionally, as only 1 isolate was stored from each *K. pneumoniae* positive infection or carriage specimen, it is possible that the proportion of matching infection/carriage pairs underestimates the contribution of carriage to infection and misses some instances of transmission.

Our conclusion that the GI microbiome is a source of *K. pneumoniae* infections in ICU patients echoes similar findings that colonizing strains of *S. aureus*, *A. baumannii*, *Enterococcus*, and Enterobacteriaceae are a common source of HA infections [12, 23–25]. Routine screening for nasal carriage of methicillin-resistant *S. aureus* or gut carriage of vancomycin-resistant *Enterococcus* or ESBL/CP Enterobacteriaceae has been introduced in various hospital settings [25–28]. A recent study of CP *K. pneumoniae* in Israel suggested screening and isolation of carriers could help end current outbreaks and prevent future ones [3]. A similar study introduced screening for ESBL *K. pneumoniae* in order to limit and prevent current and future outbreaks [29]. Although those studies focus on screening for CP or ESBL *K. pneumoniae*, our results indicate that routine screening for general *K. pneumoniae* carriage in the ICU could also be a valuable tool. Foreknowledge of the antimicrobial susceptibility profiles of *K. pneumoniae*, as well as other opportunistic pathogens resident in the microbiome, could guide the choice of prophylactic and therapeutic antimicrobial treatment [21, 22, 25].

## Supplementary Data

Supplementary materials are available at *Clinical Infectious Diseases* online. Consisting of data provided by the authors to benefit the reader, the posted materials are not copyedited and are the sole responsibility of the authors, so questions or comments should be addressed to the corresponding author.

## Supplementary Material

SupplementaryTable1

SupplementaryTable3

Supplementary_Information

## Notes

***Acknowledgments.*** The authors gratefully acknowledge the contribution and support of Janine Roney, Mellissa Bryant, Jennifer Williams, Iain Abbott and Noelene Browne at the Alfred Hospital, and the sequencing team at the Wellcome Trust Sanger Institute.

***Funding.*** This work was supported by the National Health and Medical Research Council of Australia (Project 1043822 and Fellowship 1061409 to K. E. H.) and the Australian Government Research Training Program (Scholarship to C. L. G.).

***Potential conflict of interest.*** All authors: No reported conflicts of interest. All authors have submitted the ICMJE Form for Disclosure of Potential Conflicts of Interest. Conflicts that the editors consider relevant to the content of the manuscript have been disclosed.
