# Supplementary material for: Gastrointestinal Carriage Is a Major Reservoir of Klebsiella pneumoniae Infection in Intensive Care Patients
Source: Clin Infect Dis. 2017 Mar 24;65(2):208–15. doi: 10.1093/cid/cix270 (PMC5850561; doi:10.1093/cid/cix270)
Supplement: Supplementary_Information [file cix270_suppl_supplementary_information.pdf]

SUPPLEMENTARY TABLES

Supplementary Table 1. Carriage and infection sample data and accession numbers.

[see file: SupplementaryTable1.txt]

Supplementary Table 2. Baseline GI carriage rates, with CA cohort broken down into individual days.

	**Total swabbed (n)**	**Kp positive (%)**	**Kp negative (%)**
**CA / Day 0-2**	**324**	**19 (5.9%)**	**305 (94.1%)**
- Day 0	24	1 (4%)	23 (96%)
- Day 1	206	15 (7%)	191 (93%)
- Day 2	94	3 (3%)	91 (97%)
**HA / Day 3+**	**174**	**33 (19.0%)**	**141 (81.0%)**
**Total**	**498**	**52 (10.4%)**	**446 (89.6%)**

Supplementary Table 3. Patients with time in the ICU, with carriage isolates, infection isolates, or both carriage and infection isolates.

[see file: SupplementaryTable3.txt]

SUPPLEMENTARY FIGURES

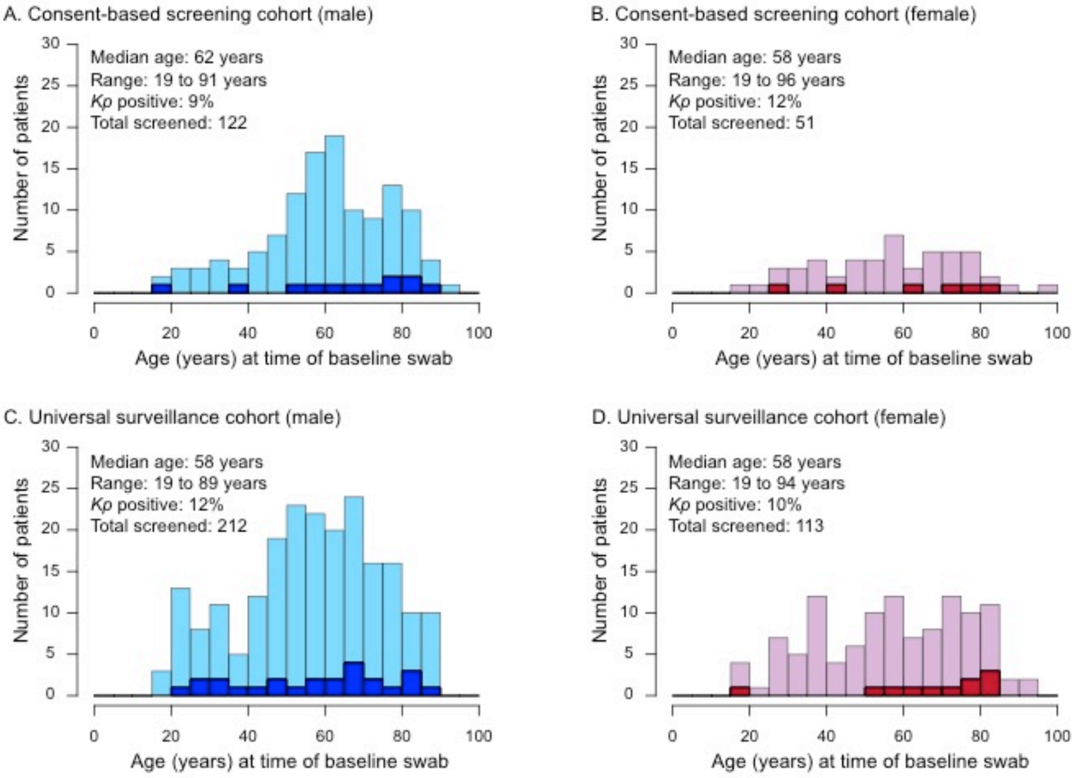

Supplementary Figure 1. Characteristics of patients by gender and recruitment period. Histograms show the distribution of ages of patients testing culture positive and negative for *K. pneumoniae*, stratified by by gender, among individuals screened for baseline carriage of *K. pneumoniae* over the first nine months (consent-based screening, panels **A** and **B**) and the final three months (universal surveillance screening, panels **C** and **D**) of the study. Lighter colours, culture negative; darker colours, culture positive.

Supplementary Figure 2. Distribution of patient ages, stratified by *Kp* culture status at baseline screening. Red line, median density; pink lines, inter-quartile ranges.

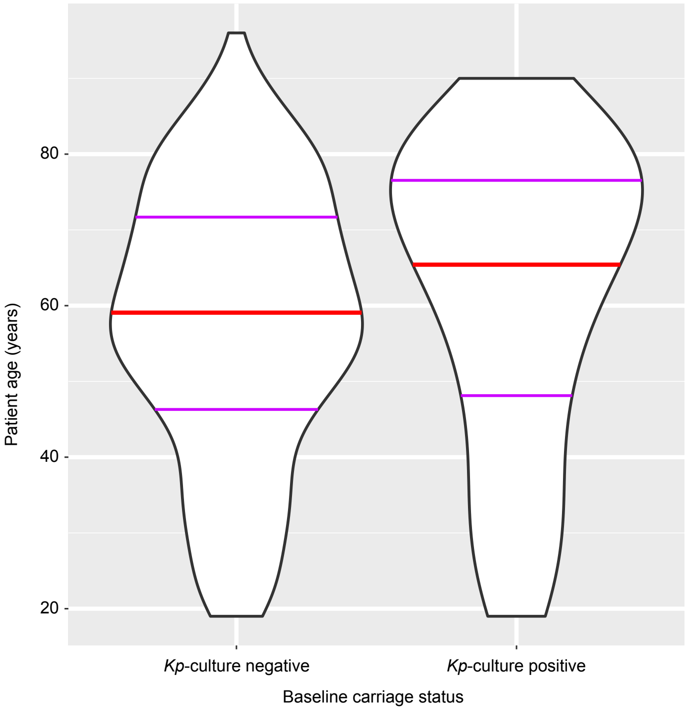

Supplementary Figure 3. Day of first swab, post admission to hospital, for individuals in the healthcare-associated (HA) group.

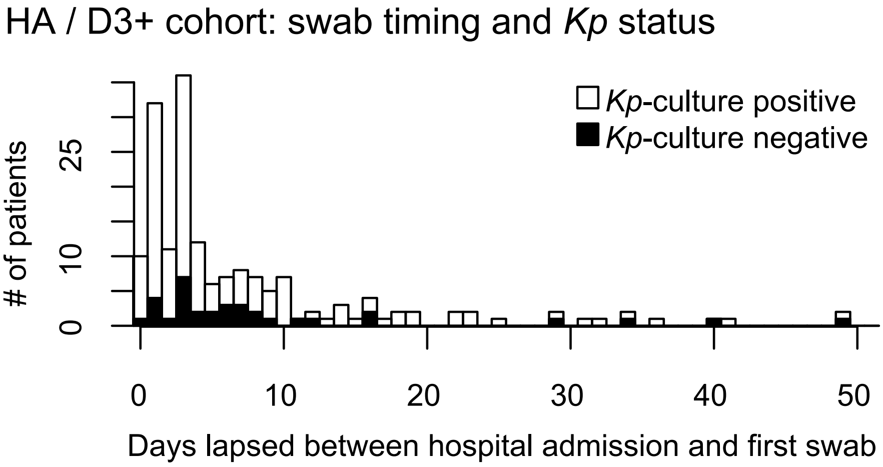

Supplementary Figure 4. Timelines for all lineages detected in multiple patients that did not show any inter-patient pairwise genetic distance between isolates of ≤25 SNPs per 5 Mbp. Lineages are boxed and labelled with their multi-locus sequence type (ST). Each horizontal dashed line indicates the time line for a patient, labelled to the left (crosses indicate date of death where applicable). Periods of Alfred Hospital admission are indicated as white boxes, periods in ICU as pink shading. Circles indicate *K. pneumoniae* infection isolates (red, belonging to the lineage; black, other lineage); triangles indicate rectal screening swabs (red, *K. pneumoniae* belonging to the lineage; black, *K. pneumoniae* of another lineage; unfilled, negative for *K. pneumoniae*). Blue boxes indicate groups of isolates for which all patients have at least one pairwise genetic distance from another in the group that falls below the cut-off indicated in the inset legend. **Clinical isolate from sputum (KC0048), no X-ray data and may represent asymptomatic colonisation.

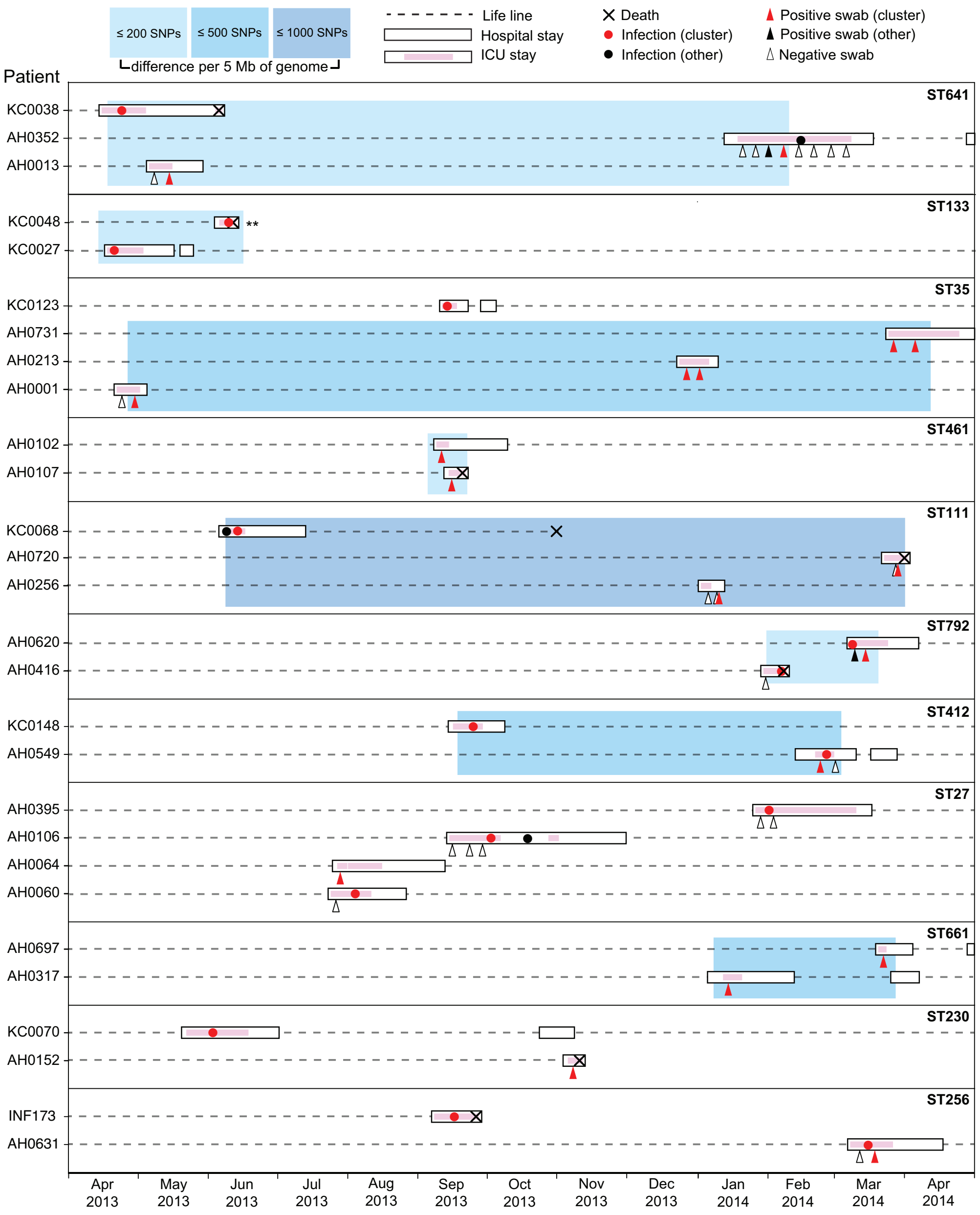

SUPPLEMENTARY METHODS

Setting

The current study, *Klebsiella* Acquisition Surveillance Project at Alfred Health (KASPAH) was conducted over a one-year period from April 1, 2013 – March 31, 2014. The study includes monitoring for all *K. pneumoniae* clinical isolates collected from patients in the Alfred Hospital, and recruitment of Alfred Hospital ICU patients for *K. pneumoniae* carriage screening, as detailed below. The Alfred Hospital is a 350-bed tertiary referral hospital that includes a 45-bed intensive care unit (ICU) with specialist cardiac and trauma services in addition to caring for general medical and surgical patients. All patients in the ICU are managed in single cubicles in 3 sub-specialised 'pods'. Each cubicle has three permanent walls with a fourth wall that may be i) open to the central area of the pod, or ii) closed by means of sliding glass doors.

Clinical isolates

Clinical isolates of *K. pneumoniae* were included when the treating physician referred a specimen to the diagnostic service of the Alfred Microbiology laboratory for analysis based on clinical suspicion of infection, and *K. pnemoniae* was subsequently identified and reported as a pathogen according to the in-house standard operating procedures. All *K. pneumoniae* identified from sterile sites (blood cultures, cerebrospinal fluid, deep tissue biopsies, pleural fluid) and from cultured prosthetic material (eg. central venous catheters) were reported as pathogens, as long as other enteric or skin flora were not detected. For other specimen types, infection was deemed present if sufficient concentrations of neutrophils were seen on microscopy or Gram stain and *K. pneumoniae* was found to be the sole organism present, or the predominant organism if the sample was expected to contain normal flora. *K. pneumoniae* would be reported as an infection in the absence of neutrophils if the patient was neutropaenic. The vast majority of isolates resulted from wound swabs, sputum samples and urine samples. Where *K. pneumoniae* is identified in urine samples or wound swabs along with other enteric bacteria (e.g. *E. coli*), the lab reports this as mixed enteric flora and *K. pneumoniae* isolated from such specimens were excluded from the study. Wound swabs were collected when signs of infection were present, i.e. purulent discharge); and reported as *K. pneumoniae* only when other enteric or skin bacteria were not also identified. Sputum samples were only sent for testing when sputum was produced by the patient (the lab rejects saliva samples received as sputum); and reported as *K. pneumoniae* when this was present in large amounts with no other pathogens detected. Case notes were reviewed to confirm that all *K. pneumoniae* positive sputum samples were accompanied by chest X-rays (23/29) and/or other clinical signs (5/29) indicative of pneumonia; 1 sputum culture-positive trauma patient (KC0048) was not subjected to X-rays or other investigations as they died shortly after due to catastrophic injuries, this ST133 *Kp* isolate may not represent a genuine infection and is indicated (**) in Figure 2 and Supplementary Figure 4. While the microbiological diagnostic laboratory at the Alfred Hospital serves the wider Alfred Health network, only clinical isolates obtained from patients at the Alfred Hospital are reported in the present study.

Recruitment into carriage study

Trained clinical research nurses directly approached patients that met the criteria for enrolment into the study. For the first nine months of the study (consent-based collection) verbal consent was requested from ICU patients aged 18 years and older who were thought likely to stay in ICU for ≥3 days. The eligible study population were patients spending 3 or more days in ICU, as we considered this group most at risk of developing *Klebsiella* colonisation and/or infection during their stay in hospital. Half of all Alfred Hospital ICU patients are admitted following scheduled heart surgery or other procedures and spend only 1-2 days there; these patients were not considered at significant risk of acquiring *Klebsiella* colonisation or infection and were not included in the study. If the patients themselves were unable to give consent then adults responsible for them were approached on their behalf. Permission was sought for a rectal and a throat swab to be taken each 5 to 7 days during the admission in ICU, and for medical information to be collected from hospital records. During this period, 33% of ICU patients spending ≥3 days in ICU were enrolled and screened for *Kp* carriage (n=174); this low recruitment rate was due to the requirement to avoid disruption to patient care, and practical issues in identifying and approaching appropriate family members or persons responsible to give informed consent to participate in the study. For the final 3 months of recruitment (universal collection), a multidrug-resistance surveillance study (#526/13) was concurrently conducted by our group in the ICU. For this surveillance study, ethical approval was granted to collect rectal swabs and patient data without the requirement for verbal consent, although patients who were conscious had the option to refuse. Throat swabs were not obtained during this period. Ethical approval was also obtained to include screening swabs and patient data collected during the surveillance study in the *K. pneumoniae* study. During this period, 75% of ICU patients spending ≥3 days in ICU were enrolled and screened for *Kp* carriage (n=324). **Supplementary Figure 1** shows the distribution of age, sex and *Kp* carriage amongst the patients recruited during the two study periods, which shows no significant differences between the two groups; therefore results were combined for all subsequent analyses.

Sample and patient data collection

Baseline

Baseline carriage swabs and accompanying patient data was collected for all study participants as soon as possible after recruitment. Each participant's medical reference number (MRN) was recorded and a study number assigned. A clinical questionnaire was then completed by nurses, based on current hospital records and charts. This questionnaire included information on age, gender, date of hospital and ICU admission, any surgery (including type) in the last 30 days, and any antibiotic treatment (including type given) in the last 7 days. A rectal swab (and in the first 9 months a throat swab) was then taken and the date recorded.

Follow-up
If patients were in the ICU for an extended period following their baseline screening swab(s), a follow-up swab was taken, typically 5-7 days after baseline. Some patients also had follow-up swabs after discharge from the ICU to another ward in the same hospital, which occurred within 5 days after the ICU discharge. A clinical questionnaire was again completed by nurses based on current hospital records and included all the same information as the baseline questionnaire. In addition to the information collected at baseline and follow-up, ICU and hospital discharge dates and, where applicable, death dates and ward transfer records were also extracted from participants' hospital records, where applicable, for all patients involved in possible transmission chains.

Screening swabs
Throat swabs were obtained using a sterile cotton swab, moistened with sterile normal saline, which was gently rolled across both palatal fauces with a mucosal contact time of 3-5 seconds. To obtain rectal swabs, a similar sterile cotton swab was moistened with sterile normal saline and then inserted into the distal rectum and gently rotated for 3 seconds. Swabs were sent to the microbiological diagnostic laboratory at the Alfred Hospital for analysis.

Each swab was plated onto MacConkey agar and incubated in air at 36° for 24 hours. The swab tip was then placed into heart infusion broth and also incubated for 24 hours. The heart infusion broth was subcultured onto a MacConkey plate and incubated in air at 36° for 24 hours. If there was no significant growth at 24 hours, plates were re-incubated until 48 hours had elapsed. Any colony with the appearance of *Klebsiella* species (convex, mucoid, lactose-fermenting (i.e. pink on MacConkey agar)) was investigated. Species identification was made using matrix-assisted laser desorption ionization-time of flight (MALDI-TOF) analysis with a Vitek MS (bioMerieux, Marcy L'Etoile, France). If the colony chosen was not *K. pneumoniae,* two further colonies were sampled for MALDI-TOF testing. If a colony resembling *K. pneumoniae* had an unconfirmed identification reported by the MALDI-TOF, further identification analysis was performed using other methods as appropriate; these included motility testing and the Vitek2 GNI card (bioMerieux, Marcy L'Etoile, France).

Antimicrobial susceptibility testing
Antimicrobial susceptibility testing was performed using the Vitek2 GNS card using breakpoints determined by EUCAST and CLSI. Antimicrobials tested were: amikacin, amoxicillin/clavulanic acid, ampicillin, cefazolin, cefepime, cefoxitin, ceftazidime, ceftriaxone, ciprofloxacin, gentamicin, meropenem, nitrofurantoin, norfloxacin. piperacillin/tazobactam, ticarcilin/clavulanic acid, tobramycin, trimethoprim, and trimethoprim/sulfamethoxazole. If the susceptibility pattern suggested an extended-spectrum beta-lactamase this was confirmed using the method of Jarlier[1]. Resistance phenotypes are reported in **Supplementary Table 1** according to CLSI guidelines. Isolates were classified as multidrug resistant (MDR) if they were insusceptible or resistant to more than two classes of antibiotics, not including ampicillin and nitrofurantoin, according to CLSI guidelines.

Community associated vs healthcare associated *K. pneumoniae* carriage

Individuals swabbed for *K. pneumoniae* carriage were separated into groups in order to obtain a clear assessment of carriage rates among individuals admitted from the general community with no recent hospital exposure. Individuals in the community associated (CA) screening group include patients who (a) were admitted to the Alfred ICU either directly (day 0), or via another ward of the Alfred Hospital on day 0, 1 or 2 of the original Alfred Hospital admission; and (b) were first swabbed for *K. pneumoniae* carriage on day 0, 1 or 2 of that admission. Individuals in the CA screening group are further stratified into Day 0, Day 1 or Day 2 according to the day on which they were first swabbed, with day 0 being the day of admission to the Alfred Hospital. All patients who were first swabbed on day 3 or later of their Alfred Hospital admission are included in the HA / Day 3+ screening group. Individuals referred to the Alfred ICU by the trauma ward of another hospital are assumed to be emergency admissions from the community, and are assigned to the CA / Day 0-2 or HA / Day 3+ screening groups according to the day of first swab relative to their Alfred Hospital admission. All other patients transferred from another hospital are included in the HA / D3+ screening group, as it is assumed that they have been exposed to a hospital setting for an undetermined period. As such, the CA / Day 0-2 screening groups represent individuals admitted directly to the hospital from the community, whereas the HA / D3+ group includes individuals with recent hospital exposure. These groups were further separated into those with and without a recorded surgery in the 30 days prior to screening. The median number of days lapsed between hospital admission and screening for the CA group is 1 day (range: 0 to 2 days), and the median for the HA group is 3 days (range: 0 to 49 days), see **Supplementary Figure 2**.

DNA extraction and sequencing

Samples were plated onto LB agar plates and incubated overnight at 37°C with air. Following this, single colonies were picked and subcultured into LB broths and again incubated overnight at 37°C with air and with agitation. Samples which had mucoid colonies – or which did not spin down during the first step of the extraction – were incubated in 10 mL Luria broths with 32-35ul of 10 mg/mL sodium salicylate (stock concentration of ~6.2nM) added to each broth, in order to inhibit capsule formation. Cells were harvested from a 5 mL overnight culture by centrifugation, then resuspended in in 2 mL PBS (pH 7.4). 250 µL of 20% SDS and 25 µL of 20 mg/mL proteinase K were added and then suspension was briefly vortexed. Following this, the tube was incubated at 37°C for 45 to 60 minutes, or until lysate cleared. Lysate was extracted once with 2 mL phenol:chloroform:isoamyl alcohol in pre-spun 15 mL light phase lock gel tube (5PRIME); samples were mixed by manual shaking until a milky emulsion formed then centrifuged for five minutes at 3750 rpm and the aqueous phase recovered into a new 15 mL light phase lock gel tube. 2 mL of chloroform:isoamyl alcohol was added then again mixed manually and centrifuged. The aqueous phase was recovered into a 10 mL tube, with 5 mL 100% ethanol and 200 µL of 2M sodium acetate (pH5.2), then gently inverted until DNA precipitated. DNA was then transferred to a 1.5 mL Eppendorf tube with 1 mL of 70% ethanol, for 5 minutes, then again transferred to a new 1.5 mL tube to air dry. The DNA was then be resuspended in 0.5 mL dH_2O or TE buffer.

Following extraction, DNA libraries were prepared using the Nextera® XT 96 barcode DNA kit, or according to the library preparation step in the Illumina sequencing protocol with some amendments[2], and sequenced on the HiSeq 2500 platform (Illumina), generating paired-end reads of 125 bp each.

Sequence analysis

A total of 94 carriage isolates and 57 infection isolates obtained from 109 patients were eligible for inclusion as the patients had spent time in the ICU. Three of these sequence data sets were excluded from further analysis, as preliminary screening showed that the sequenced colonies were dominated by non-*K. pneumoniae* DNA (two follow-up carriage isolates from two different patients were dominated by *Escherichia coli* DNA with 17-19x depth of *Klebsiella* reads, and one infection isolate was dominated by *Acinetobacter baumannii* DNA with 5x depth of *Klebsiella* reads). Note this does not mean that the original identification of *K. pneumoniae* from these samples was incorrect, but could also be explained by either a mixture of bacteria in the culture with the colony picked for subculture and DNA extraction happening to be dominated by non-*Klebsiella* cells; or a *Klebsiella* specimen becoming contaminated with non-*Klebsiella* cells or non-*Klebsiella* DNA originating from other samples. Due to the original lab identification as *Kp*, and the confirmed presence of *Kp* DNA, we therefore count these three specimens as positive detection of *Kp*; but exclude the genome data for further detailed analysis. The remaining 148 WGS-confirmed *Kp* isolates (92 carriage isolates and 56 infection) from 106 patients underwent further comparative *Kp* genomic analysis.

Multi-locus sequence typing (MLST) was performed by comparing all read sets to *K. pneumoniae* 7-locus MLST scheme[3] using SRST2 v0.2.0[4]. For core gene phylogenetic analysis, single nucleotide polymorphisms (SNPs) were identified by mapping Illumina reads against the *K. pneumoniae* strain NTUH-K2044 (ST23) reference genome, using the mapping pipeline RedDog v1b10.2 (https://github.com/katholt/reddog). RedDog uses Bowtie2 v2.2.5[5] to map reads and SamTools v1.2[6] to call SNPs with phred quality score 30, as described previously[7]. The SNP data identified five isolates with significant heterozygosity (ratio of ≥0.1 heterozygous SNP to every homozygous SNP (het/hom ratio)), originating from mixed isolates that each comprised a mixture of dominant and secondary *K. pneumoniae* clones or of a mixture of *K. pneumoniae* and non-*Klebsiella*. As their phylogenetic placement and high-resolution pairwise SNP distances could not be accurately determined, these were excluded from further analysis, leaving a total of n=143 genomes. Core genes were defined as those that were annotated in the reference genome and present (coverage ≥ 95% and depth ≥ 5x) in all of the sequenced isolates based on mapping analysis. A phylogenetic tree was inferred from an alignment of all homozygous SNPs identified within core genes (n=3,419) in the 143 genomes, using FastTree v2.1.8[8, 9] (**Figure 2**; six isolates with het/hom ratio in the range 0.02-0.1, which could indicate low-level mixed culture, are indicated with a *). Phylogenetic clusters were identified by analysing this tree using RAMI[10], with a patristic distance threshold of 0.02 (~11,800 SNPs).

Genetic distances

Isolates falling within the same phylogenetic lineage were further investigated to identify pairwise SNPs. The six isolates with het/hom ratio in the range 0.02-0.1 were excluded from this analysis, leaving 137 genomes. As the gene content of *K. pneumoniae* isolates can vary substantially[11], the amount of sequence that is alignable between pairs of *K. pneumoniae* genomes varies substantially. We therefore used a pairwise cross-mapping approach to estimate the number of SNPs between pairs of isolates, rather than using the mapping to a common reference as we had done for phylogenetic analysis. Each read set was assembled using SPAdes v3.6.2[12] with kmer lengths of 31, 41, 51, and 61 and the 'before repeat resolution' contig sequences were used for subsequent comparative analysis in order to maximize the amount of alignable sequence. For each pair of isolates, a pairwise nucleotide BLAST of contig sequences was used to identify and extract the full complement of homologous sequences between the two genome assemblies (interquartile range, 81-90% of the total assembled bases in each assembly) (code available at https://github.com/rrwick/Catpac). Isolates were further excluded at this if point if median read depth was <20 reads. For each pair of isolates, SNPs within the homologous regions were then identified by mapping both sets of read sets to both sets of homologous sequences using Bowtie2 v2.2.9[5] and calling SNPs with SamTools v1.3.1[6]. Unreliable SNP calls (defined as a mapping quality score <50, <80% of reads supporting a variant call, <5 good quality forward and reverse reads supporting the variant (as extracted from DP4 values in the VCF file), or those also called from self-mapping of reads) were filtered out. This resulted in two estimates of SNP counts for each pair of strains, A and B: those called from mapping isolate A reads to isolate B assembly, and those called by mapping isolate B reads to isolate A assembly. These counts were on average within 1.5% of one another, and the greatest of the two values was taken as a conservative estimate of pairwise SNP distance between isolates A and B. As the alignable portion of any two *K. pneumoniae* genomes varies substantially according to their relatedness, these SNP counts were normalised to the length of each pairwise alignment, yielding comparable estimates of pairwise genetic distances in terms of SNPs per base. For ease of interpretation, these pairwise distances are expressed in units of SNPs per 5 Mbp as an approximation to the total number of SNPs per genome.

Identification of transmission chains

Genetic distance cut-offs for likely and very likely strain sharing were determined by inspecting the distribution of pairwise SNP distances between isolates from the same patient with those from different patients (**Figure 3**). Comparison to dates of ICU admission and specimen isolation showed that, in all cases where between-patient genetic distances fell below these cut-offs, the patients in question had overlapping stays or were part of chains of patients with overlapping stays (**Figure 4**) and were deemed to represent epidemiologically plausible intra-hospital transmission chains. This accounted for 5/16 lineages that were detected in more than one ICU patient. The other 11/16 lineages had pairwise between-patient SNP distances exceeding the cut-offs for strain sharing and inspection of the temporal data showed they also lacked overlapping ICU admissions (**Supplementary Figure 4**). Such lineage sharing could result from independent acquisition in the community prior to hospital

admission, but could also potentially be explained by reservoirs of bacteria that persist within the hospital between admissions (for example, in reservoirs such as sinks or drains, or colonisation of healthcare workers [13-15]).

Statistical analysis

All statistical analyses were conducted using R (v3.3.1). Fisher's exact test (function *fisher.test*) was used to investigate associations expressed in 2x2 contingency tables and to calculate the corresponding odds ratios, confidence intervals and p-values (two-sided tests in all cases). The Wilcoxon rank-sum test was used to compare the distribution of ages between *Kp*-carriage positive and *Kp*-carriage negative participants.

References

1. Jarlier V, Nicolas MH, Fournier G, Philippon A. Extended broad-spectrum beta-lactamases conferring transferable resistance to newer beta-lactam agents in Enterobacteriaceae: hospital prevalence and susceptibility patterns. Rev Infect Dis **1988**; 10(4): 867-78.
2. Quail MA, Kozarewa F, Smith A, et al. A large genome center's improvements to the Illumina sequencing system. Nat Methods **2008**; 5(12): 1005-10.
3. Brisse S, Brisse C, Fevre V, et al. Virulent Clones of *Klebsiella pneumoniae*: Identification and Evolutionary Scenario Based on Genomic and Phenotypic Characterization. PLoS ONE **2009**; 4(3): e4982.
4. Inouye M, Dashnow H, Raven L-A, et al. SRST2: Rapid genomic surveillance for public health and hospital microbiology labs. Genome Med **2014**; 6(11): 1-16.
5. Langmead B, Salzberg SL. Fast gapped-read alignment with Bowtie 2. Nat Methods **2012**; 9(4): 357-9.
6. Li H, Handsaker B, Wysoker A, et al. The Sequence Alignment/Map format and SAMtools. Bioinformatics **2009**; 25(16): 2078-9.
7. Schultz MB, Thanh DP, Do Hoan NT, et al. Repeated local emergence of carbapenem-resistant Acinetobacter baumannii in a single hospital ward. Microb Genom **2016**; 2(3).
8. Price MN, Dehal PS, Arkin AP. FastTree: computing large minimum evolution trees with profiles instead of a distance matrix. Mol Biol Evol **2009**; 26(7): 1641-50.
9. Price MN, Dehal PS, Arkin AP. FastTree 2–approximately maximum-likelihood trees for large alignments. PloS One **2010**; 5(3): e9490.
10. Pommier T, Canbäck B, Lundberg P, Hagström Å, Tunlid A. RAMI: a tool for identification and characterization of phylogenetic clusters in microbial communities. Bioinformatics **2009**; 25(6): 736-42.
11. Holt K, Wertheim H, Zadoks R, et al. Genomic analysis of diversity, population structure, virulence, and antimicrobial resistance in *Klebsiella pneumoniae*, an urgent threat to public health. Proc Nat Acad Sci U S A **2015**; 112(27): E3574-E81.

12. Bankevich A, Nurk S, Antipov D, et al. SPAdes: A new genome assembly algorithm and its applications to single-cell sequencing. J Comput Biol **2012**; 19(5): 455-77.
13. Larson E. Persistent Carriage of Gram Negative Bacteria on Hands. Nursing research **1982**; 31(2): 121.
14. Krishna BVS, Patil AB, Chandrasekhar MR. Extended Spectrum β Lactamase producing *Klebsiella pneumoniae* in neonatal intensive care unit. Indian J Pediatr **2007**; 74(7): 627-30.
15. Starlander G, Melhus Å. Minor outbreak of extended-spectrum β-lactamase-producing *Klebsiella pneumoniae* in an intensive care unit due to a contaminated sink. J Hosp Inf **2012**; 82(2): 122-4.
